# Editorial: Current Progress in Mesenchymal Stem/Stromal Cell Research

**DOI:** 10.3389/fcell.2021.658903

**Published:** 2021-02-18

**Authors:** Lindolfo da Silva Meirelles, Karen Bieback, Marcela F. Bolontrade

**Affiliations:** ^1^Universidade Luterana do Brasil, Canoas, Brazil; ^2^Institute for Transfusion Medicine and Immunology, Medical Faculty Mannheim, Heidelberg University, Heidelberg, Germany; ^3^Instituto de Medicina Traslacional e Ingeniería Biomédica (IMTIB) CONICET, Instituto Universitario del Hospital Italiano, Hospital Italiano de Buenos Aires, Buenos Aires, Argentina

**Keywords:** mesenchymal stem cells, mesenchymal stromal (or stem) cells, mesenchymal stromal cells, basic research, MSC therapies

Currently, mesenchymal stem/stromal cells (MSCs) are among the most used cell types in clinical trials. At the time this Editorial was written (mid-January 2021), a search at www.clinicaltrials.gov using the term “MSCs OR mesenchymal stem cells OR mesenchymal stromal cells” returned 10,407 registered clinical trials using these cells, 3,467 of which were active. While many characteristics of these cells have been described to date, aspects of their basic biology that may have an impact on their use in regenerative medicine are still under investigation. Likewise, in spite of the successful use of these cells in tissue engineering and cell therapy scenarios, further work on ways to enhance their aptitude for therapeutic purposes are still under development.

The aim of this Research Topic was to provide a venue for high-quality research articles and reviews that could broaden the knowledge on the biology of mesenchymal stem/stromal cells, including mechanisms that can affect their role as agents in cell therapy and tissue engineering. Manuscripts submitted to this Research Topic were expected to contain information to better understand how MSCs behave, and how they exert their therapeutic properties, whether such information would come from *in vitro* or *in vivo* studies. Consequently, suggested subjects for submitted manuscripts included (a) novel features of MSCs from different tissues; (b) molecular aspects of MSC differentiation; (c) processes for the enhancement of MSC differentiation and expansion in culture; (d) identification of MSCs, or cells that can give rise to them, *in situ*; (e) acellular MSC-derived products such as extracellular vesicles; (f) immunomodulatory properties of MSCs; (g) molecular interactions between MSCs and other cell types *in vitro* or *in vivo*; and (h) reviews of achievements and challenges in translating MSCs from the bench to bedside, including the development of authorized advanced therapy medicinal products. Consequently, this Research Topic received 26 manuscripts. Seventeen of these manuscripts were accepted; eight of these were original research reports, and nine were reviews or mini-reviews.

The review articles in this Research Topic cover a wide range of subjects in the MSC field. The review by Bozorgmehr et al. highlights biological properties and clinical applications of endometrial and menstrual blood MSCs, which comprise cell populations that share many characteristics with MSCs obtained from other tissues. Robert et al. review the transcriptomic changes that take place during adipogenic, osteogenic, and chondrogenic differentiation of MSCs from various body sites. Kamdje et al. review the ability of the tumor microenvironment to reprogram MSCs toward a phenotype that favors tumoral maintenance. Forsberg et al. addresses the development of MSC-based treatments for human diseases, particularly treatments using MSC-derived exosomes, in contexts like acute radiation syndrome, graft vs. host disease, cardiovascular conditions, and COVID-19. Daltro et al. introduce and review the concept of using MSCs for the treatment of inflammatory/allergic skin disorders such as atopic dermatitis, and suggest that the broad immunomodulatory potential of MSCs on T cells, B cells, and mast cells may prove beneficial in these cases. Damasceno et al. review whether and how genetic engineering can be applied to improve the therapeutic efficacy of MSCs. They summarize the current knowledge on using viral vectors and non-viral methods to express bioactive molecules in the context of cancer treatment and various diseases. Kamal and Kassem review achievements, as well as challenges/hurdles in establishing Wharton's jelly-derived MSCs as therapeutic agents for the treatment of diabetic complications while focusing on the potential of these cells to home and differentiate/replace diseased cells, and to secrete bioactive factors that can modulate immune responses; additionally, these authors stress existing challenges to translate this knowledge from the bench to the bedside. Lavrentieva et al. review possible pitfalls in the production of human MSCs for cell therapies, focusing on the heterogeneity of current strategies used to obtain MSCs for clinical applications. Finally, Ramezankhani et al. summarize authorized advanced therapy medicinal products (ATMPs) currently available and review accomplishments, clinical trial safety, and efficacy data available for various ATMPs during the last two decades. They introduce a classification of the approved ATMPs, providing descriptions together with manufacturer, indication, approval date, regulatory agency, dosage, description, and price data.

Among the original research articles in this Research Topic, two provide aspects of the production of MSCs or extracellular vesicles secreted by them. Stroncek et al. report the variability MSC features when these cells are manufactured using different methods and source materials. Thus, they assess the impact on MSC characteristics when different laboratories propagated MSCs from cultures initiated with bone marrow aliquots derived from the same donor source material. Fuzeta et al. describes a xeno-free microcarrier-based culture system using a vertical-wheel bioreactor and a human platelet lysate culture supplement to achieve scalable production of extracellular vesicles by MSCs obtained from human bone marrow, adipose tissue, and umbilical cord matrix.

The promotion of angiogenesis by MSCs is the focus of two of the original research articles accepted. Kremer et al. compares the angiogenic potential of retinal pericytes and adipose tissue MSCs (ASCs) using angiogenesis assays in addition to transcriptomic and multiplex cytokine analyses; they found that ASCs were able to promote angiogenesis mainly through secretion of vascular-endothelial growth factor, while retinal pericytes did not exhibit pro-angiogenic properties. Whereas, Santos et al. describes the augmentation of the angiogenic potential of bone marrow MSCs by genetically modifying these cells to overexpress leukemia inhibitory factor prior to angiogenesis assays *in vitro* and *in vivo*.

Finally, four original research articles describe findings related to MSC differentiation. Díaz-Flores et al. provide an account of immunohistochemical analyses on archived samples from cases of acute cholecystitis, malignant colonic polyps, and colon adenocarcinomas; their results suggest that the development of myofibroblasts in these samples is linked to the presence of CD34-positive stromal cells. Driesen et al. identify the expression of fibroblast activation protein-α (FAPα) in human dental tissue stem cells, which are an MSC-related cell type. The findings by these authors indicate a relationship of the expression of FAPα with extracellular matrix remodeling and osteoblast function. Dilger et al. describe gap junction-dependent cell-cell communication and the expression pattern of gap junction-building connexins in MSCs during a neuronal differentiation protocol using small molecules, thus providing an account of the generation of immature neuron-like cells that may be of potential interest to explore mechanisms of MSC differentiation. Ji et al. address the mechanism of action of hsa_circ_0026827, demonstrating that it promotes osteoblast differentiation of human dental pulp stem cells via Beclin1 and the RUNX1 signaling pathways, by sponging miR-188-3p.

The fact that so many questions remain open in the MSC research field after decades of development is impressive. The ultimate goal—the widespread therapeutic use of these cells or their acellular products in the clinic—seems to be closer every day, as new clinical trials involving MSCs keep emerging. To achieve this ultimate goal, furthering the knowledge on the mechanisms underlying their potential therapeutic actions is of paramount importance, along with the clinical translation of such knowledge.

## Author Contributions

All authors listed have made a substantial, direct and intellectual contribution to the work, and approved it for publication.

## Conflict of Interest

The authors declare that the research was conducted in the absence of any commercial or financial relationships that could be construed as a potential conflict of interest.

